# Clonal structure, stability and dynamics of human memory B cells and circulating plasmablasts

**DOI:** 10.1038/s41590-022-01230-1

**Published:** 2022-06-27

**Authors:** Ganesh E. Phad, Dora Pinto, Mathilde Foglierini, Murodzhon Akhmedov, Riccardo L. Rossi, Emilia Malvicini, Antonino Cassotta, Chiara Silacci Fregni, Ludovica Bruno, Federica Sallusto, Antonio Lanzavecchia

**Affiliations:** 1 Institute for Research in Biomedicine, Università della Svizzera italiana, Bellinzona, Switzerland; 2 Bigomics Analytics SA, Bellinzona, Switzerland; 3 National Institute of Molecular Genetics, Milano, Italy; 4 Institute of Microbiology, ETH Zürich, Zurich, Switzerland; 5 Humabs BioMed, a subsidiary of Vir Biotechnology, Bellinzona, Switzerland

**Keywords:** Antibodies, Immunogenetics, Class switch recombination

## Abstract

Memory B cells persist for a lifetime and rapidly differentiate into antibody-producing plasmablasts and plasma cells upon antigen re-encounter. The clonal relationship and evolution of memory B cells and circulating plasmablasts is not well understood. Using single-cell sequencing combined with isolation of specific antibodies, we found that in two healthy donors, the memory B cell repertoire was dominated by large IgM, IgA and IgG2 clonal families, whereas IgG1 families, including those specific for recall antigens, were of small size. Analysis of multiyear samples demonstrated stability of memory B cell clonal families and revealed that a large fraction of recently generated plasmablasts was derived from long-term memory B cell families and was found recurrently. Collectively, this study provides a systematic description of the structure, stability and dynamics of the human memory B cell pool and suggests that memory B cells may be active at any time point in the generation of plasmablasts.

## Abstract (web summary)

Lanzavecchia and colleagues use single-cell B cell receptor sequencing to examine the clonal structure, stability and dynamics of the B cell repertoire and the relationship between memory B cells and newly generated plasma cells in humans.

## Main

During B cell development and antigen-driven selection, a coordinated series of recombination events and somatic mutations generate an extraordinarily large repertoire of antibody molecules that are selected for their capacity to bind to self and foreign antigens and for different effector functions^1^. In recent years, high-throughput DNA-sequencing technologies and new bioinformatics approaches have been used to dissect the diversity of antibody repertoires to an unprecedented level^2^. Two recent studies used deep sequencing of unpaired H and L chains to estimate the size and diversity of the human B cell receptor (BCR) repertoire and to identify shared VH sequences among different individuals^3,4^. In addition, bulk VH sequencing was used to characterize antigen-specific memory B cells and plasmablasts in response to infectious agents and self antigens^5–7^. Single-cell sequencing methods, although limited by the number of cells that can be processed, can deliver paired VH/VL sequences together with information on the transcriptome and have been used to dissect the antibody response to infection or vaccination^8,9^.

In this study, we took a systematic approach and used a single-cell sequencing platform combined with the isolation of specific antibodies from memory B cells and circulating plasmablasts to investigate, in multiyear serial blood samples from two healthy adult donors, the clonal structure, stability and dynamics of the entire human memory B cell repertoire and the relationship between memory B cells and circulating plasmablasts.

## Results

### Clonal structure, isotype distribution and convergent clonotypes in memory B cells

**Fig. 1:**
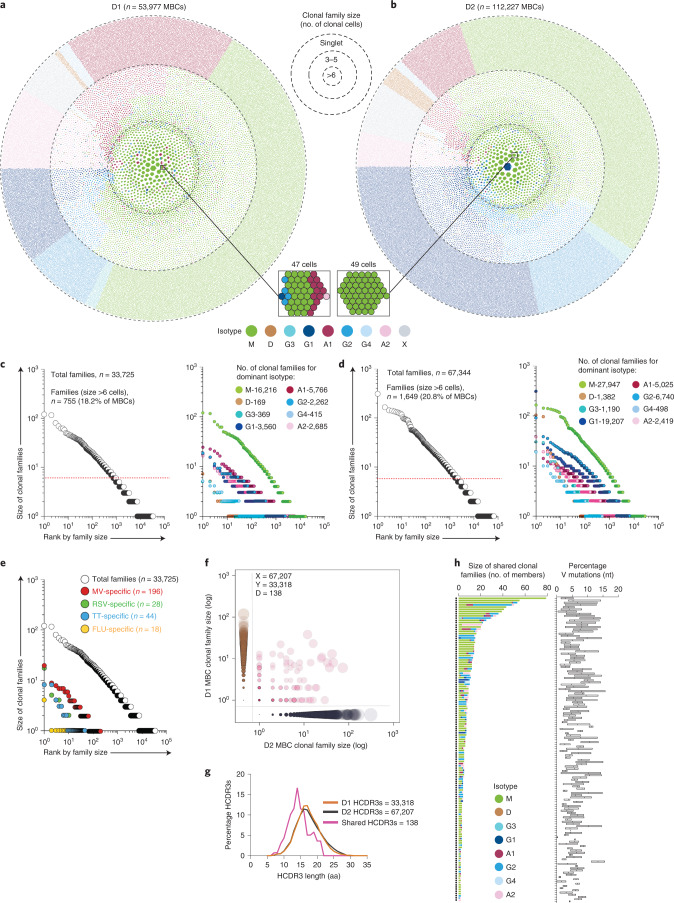
Clonal structure of the memory B cell pool and convergent antibodies. **a**,**b**, Honeycomb plots compile all memory B cells (MBCs) from D1 and D2 samples analyzed using a donor-specific database into clonal families; each cell is color-coded according to the isotype expressed and families are ranked according to size from center to periphery. Two representative clonal families comprising multiple or unique isotypes are highlighted. **c**,**d**, Waterfall plots represent the size distribution of all clonal families (left) and of families grouped according to the dominant (≥80% of cells) isotype (right) in D1 (**c**) and D2 (**d**). Dotted red lines separate families with more than six and six or fewer cells. Isotypes are color coded (x indicates isotype not determined). **e**, Size distribution of MBC families from D1 specific for the recall antigens measles virus (MV), respiratory syncytial virus (RSV), tetanus toxoid (TT) and influenza virus (FLU) determined by searching 328 antigen-specific antibody sequences (belonging to 286 clonal families) in the 10X Genomics database from D1. Size distribution of total MBC clonal families is included for comparison. **f**, Scatter-plot shows convergent clonotypes determined by jointly analyzing MBC sequences from D1 and D2 using publicly available reference sequences from the IMGT database, clonotypes shared between D1 and D2 (diagonal) and non-shared clonotypes (*x* and *y* axes) are ranked according to clonal family size. **g**, HCDR3 length distribution in shared and non-shared MBC clonotypes. **h**, Isotype usage and percent VH mutations in the shared clonotypes.

**Extended Data Fig. 1:**
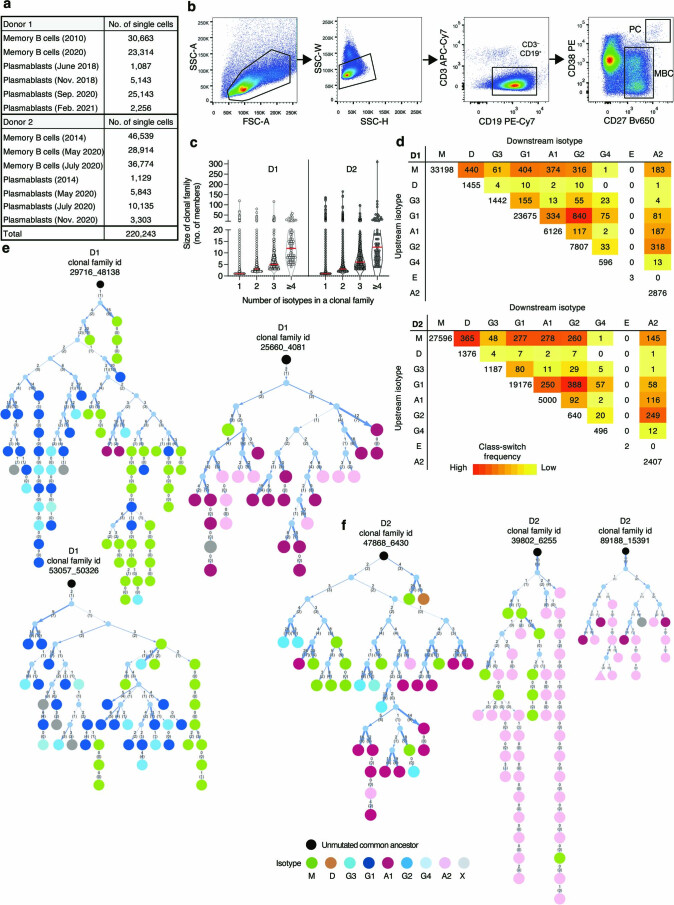
Isotype distribution and switching in memory B cell clonal families. **a**, The table shows the number of single memory B cells (MBCs) and plasmablasts (PBs) profiled from different time points from D1 and D2. **b**, Representative flow cytometry plots showing gating strategy for MBC and PB populations. **c**, Size distribution of MBC clonal families in D1 and D2 comprising one or more isotypes. Red lines show median values. **d**, Class-switch propensity matrices show the number of clonal families having clones of each possible upstream and downstream isotype pair. The number on the diagonal shows the families having a single isotype (not colored). **e,f**, Representative genealogic trees depicting the class-switching in clonal families from D1 and D2. The number of somatic mutations at nucleotide and at amino acid (in parenthesis) levels are indicated on individual branches of the trees. The vertical thin dotted lines without arrowheads connect cells with identical VH/VL sequences. The thickness of the arrows reflects the number of mutations. The order of isotypes in the branches of the family trees does not represent class-switching sequence. A comprehensive set can be found in the RepSeq Playground.

**Extended Data Fig. 2:**
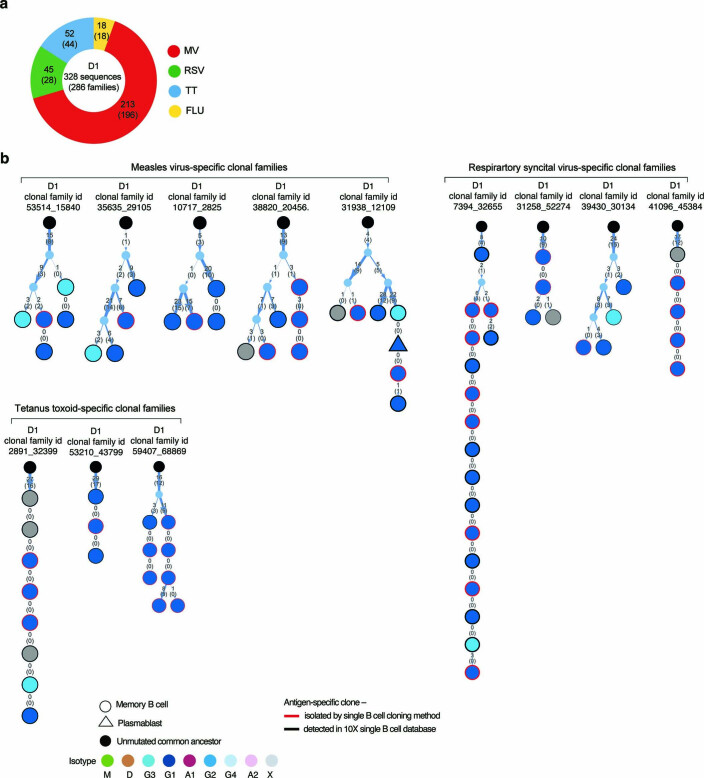
Recall antigen-specific clonal families. **a**, Pie chart shows the number of antigen-specific MBC sequences (in parenthesis, clonal families) from D1 identified through the isolation of monoclonal antibodies against influenza virus (FLU), measles virus (MV), respiratory syncytial virus (RSV), and tetanus toxoid (TT). **b**, Genealogical trees show representative recall antigen-specific clonal families in D1. Isotypes are color-coded (x=isotype not determined). Somatic mutations at nucleotide and at amino acid (in parenthesis) levels is indicated on individual branches of the trees. The vertical thin dotted lines without arrowheads connect cells with identical VH/VL sequence. The thickness of the arrows reflects the number of mutations. More examples can be found in the RepSeq Playground.

**Extended Data Fig. 3:**
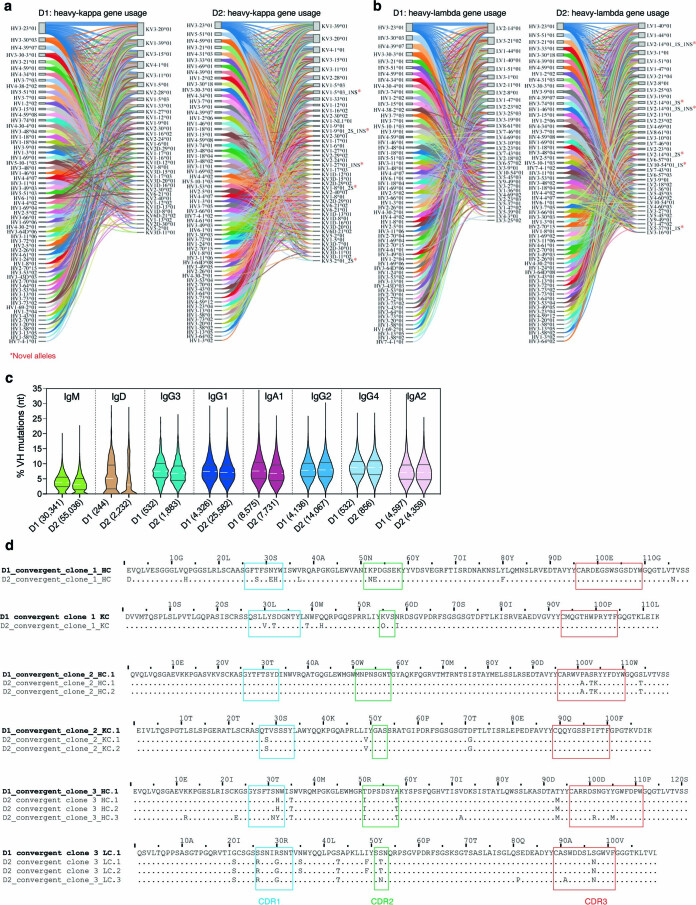
V gene usage, VH/VL pairing and load of somatic mutations. **a,b**, Gene usage and VH/VL pairing in memory B cells from D1 and D2. VH and VL genes are ranked according to their relative expression. **c**, Percent VH nucleotide mutations in memory B cells of different isotypes from D1 and D2. Number of cells analyzed is shown in parentheses. **d**, Representative amino acid sequence alignments of heavy and light chains of convergent antibodies in D1 and D2.

To gain insight into the clonal composition of the human memory B cell pool, we used the high-throughput single-cell 10X Genomics platform to obtain >2.2 × 10^5^ paired VH/VL sequences from circulating memory B cells and plasmablasts collected from two healthy adult individuals (D1 and D2) over a period of 10 and 6 years, respectively (Extended Data Fig. 1a,b). Using established bioinformatics methods, we determined the individual’s germline VH and VL genes, used paired VH/VL sequences to define clonal families and reconstructed genealogical trees^10–14^. Clonal families showed a characteristic wide range of size distribution and isotype usage, as visualized in the honeycomb plots (Fig. 1a,b). Overall, the clonal structure of the memory B cell pool was comparable in the two donors, apart from a prevalence of IgA in D1 and IgG in D2. Notably, the largest families of more than six cells accounted for 18.2% (D1) and 20.8% (D2) of total memory B cells and were primarily or exclusively IgM, followed by IgA and IgG2, whereas IgG1, IgG3 and IgG4 families were mostly of small size (Fig. 1c,d). Rare IgD families were present in both individuals, whereas IgE memory B cells were undetectable. Clonal families comprising multiple isotypes were prominent, with a clear trend for IgM + IgG2 or IgM + IgA and IgA1 + IgA2 (Extended Data Fig. 1c–f and RepSeq Playground). By searching the 10X database of D1 for VH/VL sequences of 328 virus- or vaccine-specific IgG antibodies (belonging to 286 clonal families) isolated from immortalized memory B cells of the same donor (Extended Data Fig. 2a), we determined that the corresponding IgG families were mostly of small size (Fig. 1e and Extended Data Fig. 2b). The VH, Vκ and Vλ gene usage was comparable between the two donors, with no evidence for preferential pairing between VH and VL, whereas the load of somatic mutations was variable, being lowest in IgM and highest in IgD and IgG4 memory B cells (Extended Data Fig. 3a–c). Notably, 138 clonal families (0.2% and 0.4% in D1 and D2, respectively) showed highly similar sequences in the two donors based on the strict criteria of identical V and J usage in both heavy and light chains, CDR3s of the same length and >85% nucleotide identity (Fig. 1f and Extended Data Fig. 3d). Several of these families were IgM, IgG2 and IgA, had short HCDR3, were of large size and carried variable levels of somatic mutations (Fig. 1g,h).

Collectively, this analysis shows that the memory B cell repertoire is dominated by large IgM, IgA and IgG2 families that include convergent clonotypes, whereas typical T cell-dependent IgG1 responses have a smaller footprint.

### Stability of memory B cell clonal families

**Fig. 2:**
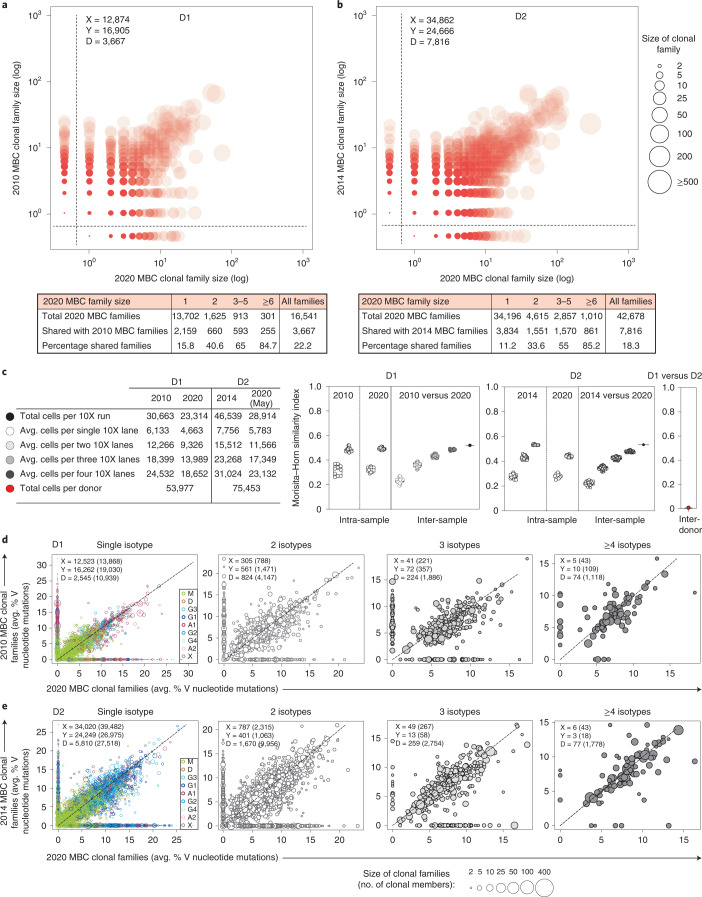
Stability of memory B cell repertoires. **a**,**b**, Scatter-plots show the projection of MBC clonal families identified in the 2020 samples (*x axis*) from D1 or D2 on MBC clonal families identified in samples collected in 2010 (D1) or 2014 (D2) (*y axis*). Shared (diagonal) and non-shared (*x* and *y* axes) MBC families ranked according to size. The size of the bubbles represents the number of members in each family. Shade of color reflects the proportion of families shared between the two time points. The tables below show the total number of 2020 MBC clonal families binned according to family size and the number and percentage of families shared with samples isolated 10 (D1) or 6 (D2) years before. **c**, Morisita–Horn similarity index calculated between small and large samples of MBCs (as indicated on the table, left) taken at the same time (intra-sample similarity) or at a different time point (inter-sample similarity) from D1 and D2. Box whisker plots with all points show the median (horizontal line), 25th–75th percentile (box), with whiskers indicating minimum and maximum range. **d**,**e**, Scatter-plots with 1:1 dashed line show shared (diagonal) and non-shared (*x* and *y* axes) MBC clonal families ranked according to average percentage V region nucleotide mutations. Clonal families are shown with circles proportional to their size and are grouped in different scatter-plots according to the expression of single isotypes (color-coded) or multiple isotypes. The number of shared and non-shared families and the number of sequences (in parenthesis) are indicated in each plot. Note that the percentage of clonal reads (B cell clones) shared between the two time points exceeds the percent of clonal families shared.

**Extended Data Fig. 4:**
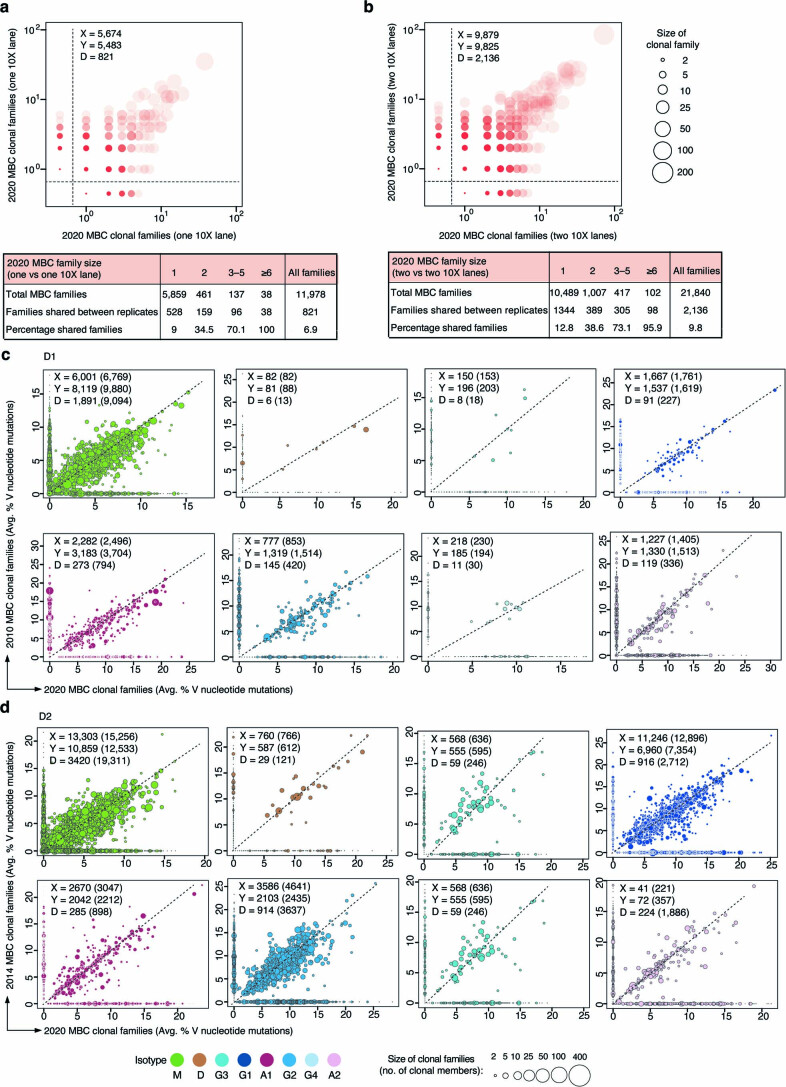
Sharing of clonal families between biological replicates collected at the same time and stability of memory B cell families of single isotypes. **a,b**, Scatter-plots show the shared (diagonal) and non-shared (xy-axis) memory B cell (MBC) clonal families from replicate samples from the same time point. Memory B cells from one vs one lanes (right) or two vs two lanes (left) of 10X chip were used to determine the sharing of families within the same sample from D2. The size of the bubbles represents the number of members in each family. Shade of color reflects the proportion of families shared between the replicate samples. Clonal family sizes were binned according to the number of members in each family in the tables shown below. The total number of MBC clonal families as well as the number and the percent of families shared between the replicate samples is shown in the table. **c,d**, Scatter-plots with 1:1 dashed line show shared (diagonal) and non-shared (*x* and *y* axes) MBC clonal families from D1 (**c**) and D2 (**d**) ranked according to average percentage V region nucleotide mutation. Clonal families are shown with circles proportional to their size and are grouped in different scatter-plots according to the expression of different isotypes. The number of shared and unshared families and the number of sequences (in parentheses) are indicated in each plot.

The availability of longitudinal samples collected with an interval of several years was instrumental to investigate the stability of the memory B cell repertoire as defined by the sharing of B cell clonal families in two temporally distant samples. As shown in Fig. 2a,b, most families of medium/large size (more than six members) found in the 2020 samples from D1 and D2 were already present in samples collected 10 and 6 years before (84.7% and 85.2% in D1 and D2, respectively). This figure dropped to 40.6% and 33.6% for families of two members and 15.8% and 11.2% for families of one member only. Of note, the decrease in the percentage of shared families as a function of their size was also evident when comparing two biological replicates taken at the same time and processed in parallel (Extended Data Fig. 4a,b).

To account for the differences in family size, we calculated the Morisita–Horn similarity index for samples taken at the same time (intra-sample similarity) or at different times (inter-sample similarity) (Fig. 2c). As expected, the index was proportional to the number of cells analyzed, but the intra-sample and inter-sample indexes were comparable, supporting, with a statistical method, the substantial stability of clonal families in the memory B cell pool.

We also compared the two time points considering for each family the load of mutations and the size and presence of multiple isotypes. According to these criteria, the extent of sharing was most prominent for large families and for those with higher load of mutations and multiple isotypes (Fig. 2d,e). Stability was also particularly evident for large IgM and IgG2 families (Extended Data Fig. 4c,d). Collectively, the extent of sharing of clonal families supports the notion of an overall stability of memory B cell clonal families over several years.

### Clonal composition of circulating plasmablasts and relationship to memory B cells

A widely held view is that circulating plasmablasts derive from ongoing immune responses to antigens and are short-lived unless they reach survival niches in the bone marrow^15^. It was therefore interesting to analyze, in the blood samples collected from the two donors, the clonal composition of plasmablasts and their relationship to memory B cells.

**Fig. 3:**
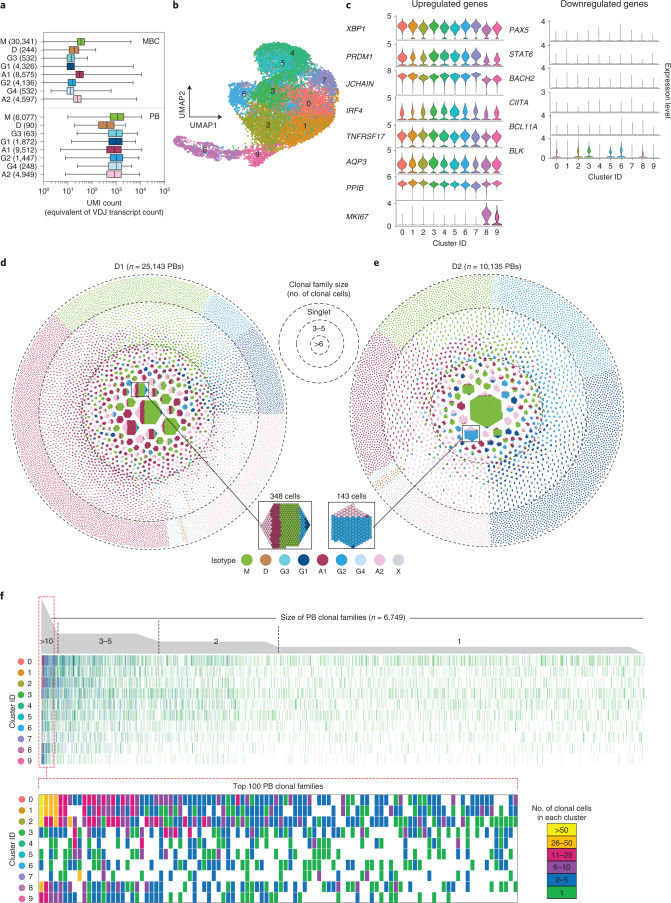
Characterization of circulating steady state plasmablasts according to transcriptome and clonotype. **a**, Level of BCR transcripts in MBCs and in circulating steady state plasmablasts (PBs) of different isotypes from D1. Comparable transcriptomic data were obtained from D2. Box whisker plots show the median (horizontal line), 25th–75th percentile (box), with whiskers indicating minimum and maximum range. **b**, Two-dimensional UMAP projection of single-cell gene expression data of circulating plasmablasts from D1. **c**, Expression of typical marker genes of plasmablasts in the different UMAP clusters. **d**,**e**, Honeycomb plots compile steady state PBs from D1 and D2 into clonal families; each cell is color-coded according to the isotype expressed. Families are ranked according to size from center to periphery. Two representative multi-isotype clonal families are highlighted. **f**, Distribution of individual cells belonging to a given clonal family in different UMAP clusters. Each column represents a PB clonal family. Shown are all families (top) and the top 100 families (bottom).

CD19^+^CD27^hi^CD38^+^HLA-DR^+^ and DR^–^ cells were isolated and subjected to 10X single-cell VDJ and transcriptomic analysis. Plasmablasts could be readily identified by the high levels of Ig transcripts, exceeding by 100-fold those found in memory B cells (Fig. 3a). Uniform Manifold Approximation and Projection (UMAP) analysis identified nine clusters all expressing high levels of plasmablast lineage markers (such as *XBP1*, *PRDM1* and *JCHAIN*) to a similar extent, and low levels of *PAX5* and *CIITA*, with clusters 8 and 9 additionally expressing the proliferation-associated gene *MKI67* encoding Ki67 (Fig. 3b,c). Notably, both HLA-DR^+^ and HLA-DR^–^ subsets were comparable in terms of the transcriptome and overlapped in their clonotypes.

**Extended Data Fig. 5:**
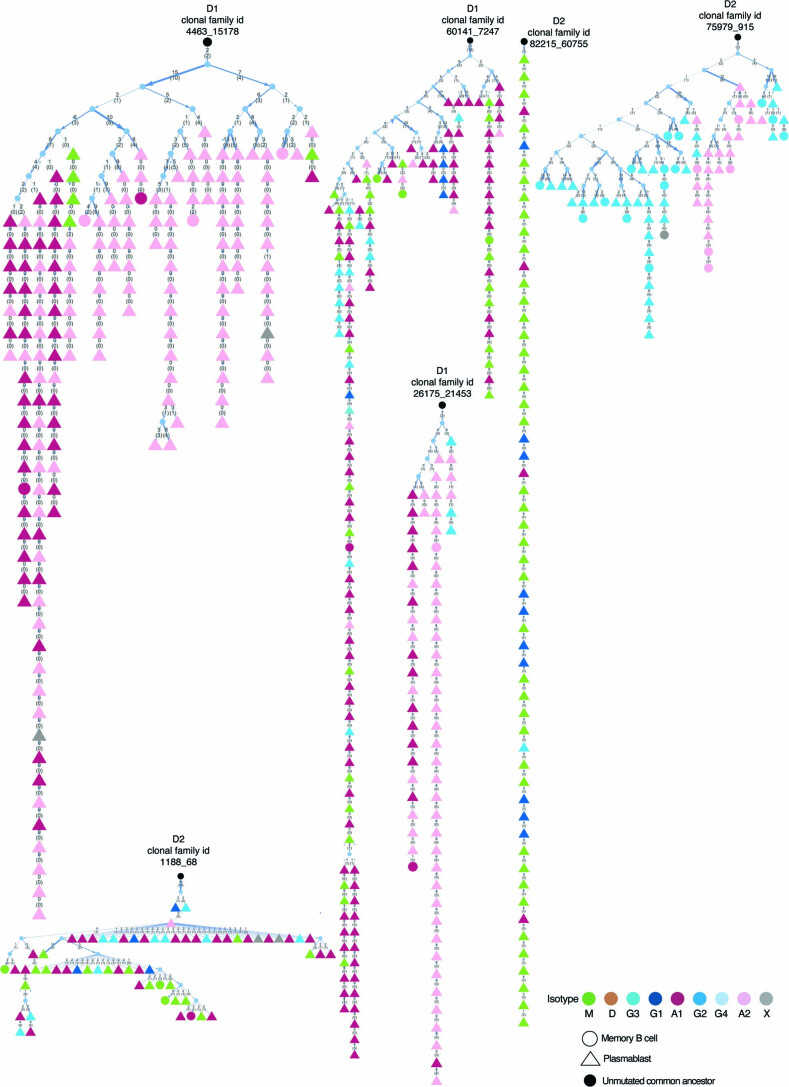
Plasmablast families comprise multi-isotype cells. Genealogical trees show representative clonal families in D1 and D2 containing multi-isotype plasmablasts (triangles). Memory B cells belonging to the same families are also shown (circles). Isotypes are color-coded (x=isotype not determined). Somatic mutations at nucleotide and at amino acid (in parenthesis) levels is indicated on individual branches of the trees. The vertical thin dotted lines without arrowheads connect cells with identical VH/VL sequence. The thickness of the arrows reflects the number of mutations. The order of isotypes in the branches of the family trees does not represent class-switching sequence. More examples can be found in the RepSeq Playground.

**Extended Data Fig. 6:**
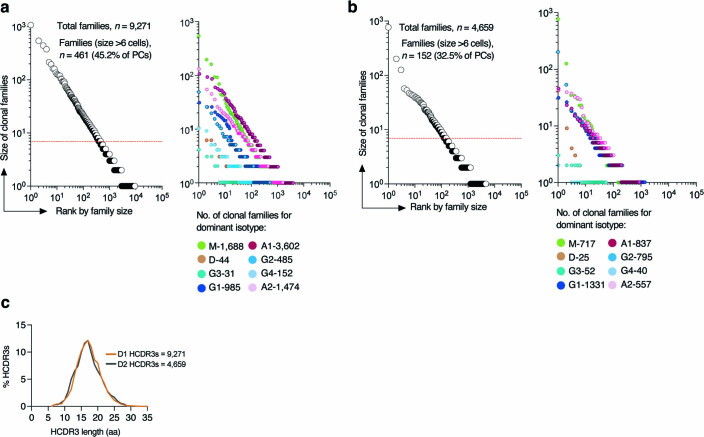
Clonal structure of circulating steady state plasmablasts. **a,b**, Waterfall plots represent the size distribution of all clonal families (left panel) and of families grouped according to the dominant (≥80% of cells) isotype (right panel) in D1 (**a**) and D2 (**b**). Dotted red line separates families with >6 or ≤6 cells. **c**, HCDR3 length distribution in plasmablasts from D1 and D2.

VH/VL gene sequencing showed that circulating plasmablasts comprised in both donors a core of highly expanded clonal families made up of several hundred cells, each with identical or related sequences (Fig. 3d,e). Notably, the most prominent clonal families were IgM, IgA or IgG2, and several contained mixed isotypes (Extended Data Fig. 5). Smaller clonal families (2–5 cells) and large numbers of singlets were also detected, possibly reflecting differences in kinetics, or burst size. The overall clonal family size distribution and CDRH3 lengths were comparable in the two donors (Extended Data Fig. 6a–c). IgA plasmablasts were more abundant in D1 and IgG plasmablasts in D2, consistent with the higher frequency of these isotypes in the memory B cell pool of the same donor. By matching VH/VL sequencing with transcriptomic data, we found that individual plasmablasts within each clonal family were distributed in different UMAP clusters, including clusters 8 and 9 expressing *MKI67* (Fig. 3f). Together with the homogeneous expression of signature genes these findings point to a microheterogeneity of circulating plasmablasts whose significance remains undefined.

**Fig. 4:**
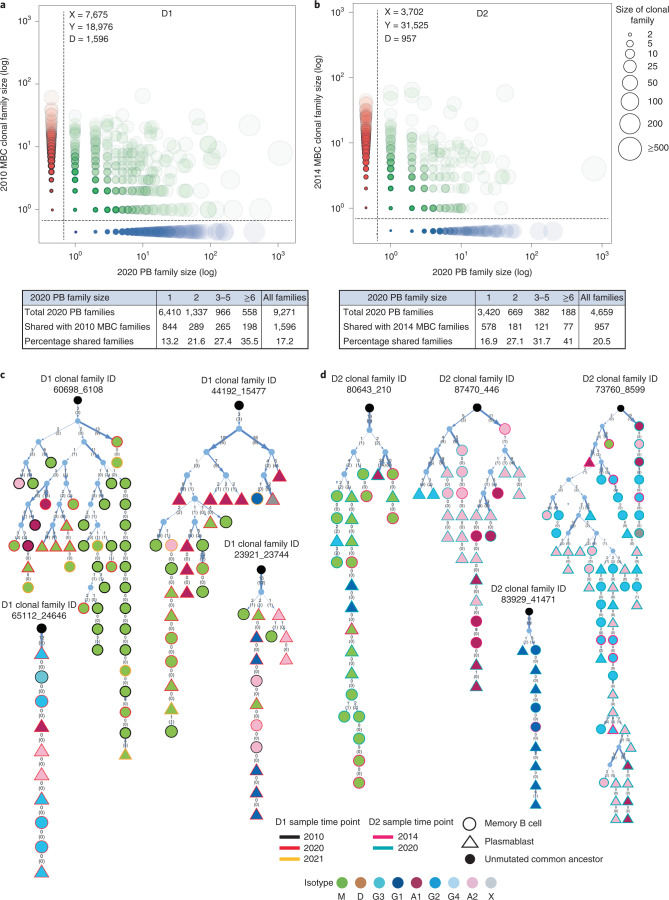
Relationship of circulating plasmablasts to long-term memory B cells. **a**,**b**, Scatter-plots show the projection of circulating plasmablast (PB) families identified in the 2020 samples (*x axis*) from D1 or D2 on MBC families identified in samples collected in 2010 (D1) or 2014 (D2) (*y axis*). Shared (diagonal) and non-shared (*x* and *y* axes) families are ranked according to size. The size of the bubbles represents the number of members in each family. Shade of color reflects the proportion of families shared between the two time points. The tables below show the total number of 2020 PB clonal families binned according to family size and the number and percent of families shared with MBC families isolated 10 (D1) or 6 (D2) years before. **c**,**d**, Genealogical trees compile PBs (triangles) and MBCs (circles) obtained at the indicated time points for six representative clonal families of D1 and D2. Isotypes are color-coded (x indicates isotype not determined). The number of somatic mutations at nucleotide and amino acid (in parenthesis) are indicated on individual branches of the trees. The vertical thin dotted lines without arrowheads connect cells with identical VH/VL sequences. The thickness of the arrows reflects the number of mutations. The order of isotypes in the branches of the family trees does not represent class-switching sequence.

**Extended Data Fig. 7:**
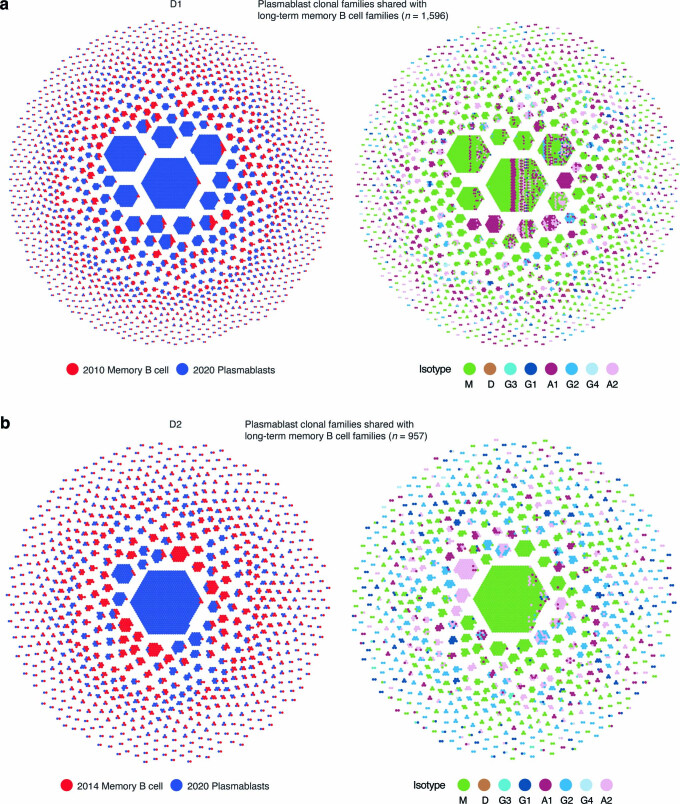
A large fraction of circulating plasmablasts derives from memory B cell clonal families identified several years before. **a,b**, The honeycomb plots on the left show the clonal relationship between circulating plasmablasts isolated in 2020 (blue) and memory B cells (red) isolated 10 (D1) and 6 (D2) years before. The honeycomb plots on the right show the isotype of the plasmablasts or memory B cells for each position.

The notion that circulating plasmablasts are generated from synchronous antigen-driven responses predicts that at any time point only a small fraction of plasmablasts should derive from memory B cells. Of note, however, 17.2% and 20.5% of all plasmablast clonal families isolated from D1 or D2 in 2020 belonged to memory B cell clonal families identified in 2010 (D1) or 2014 (D2) (Fig. 4a,b). Furthermore, genealogical analysis of individual families comprising memory B cells and plasmablasts revealed that the latter can derive from multiple branches of the tree and can express different isotypes associated with identical mutated VH/VL sequences (Fig. 4c,d, Extended Data Fig. 7 and RepSeq Playground), a finding that indicates continuous switching after somatic mutation. Collectively, the finding that at any given time point ~20% of plasmablast families derive from long-term memory B cell families indicates a continuous and substantial differentiation of memory B cells into plasmablasts and suggests that recurrent clonotypes may be found among recently generated plasmablasts.

### Recurrent clonotypes in circulating plasmablasts

**Fig. 5:**
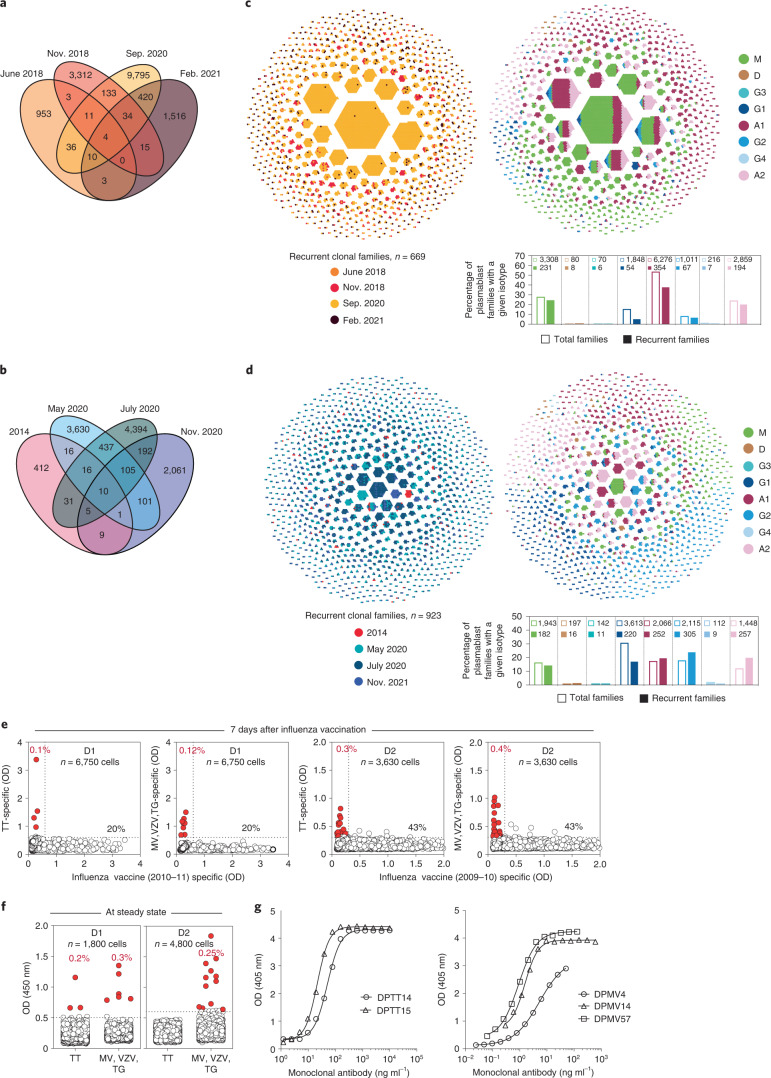
Plasmablasts in the steady state contain unique and recurrent clonotypes. **a**,**b**, Venn diagram depicts PB families shared between samples from D1 and D2 collected at the indicated time points. **c**,**d**, Honeycomb plots compile recurrent PB sequences from samples of different time points from D1 and D2 into clonal families (left) and corresponding isotype expressed by each cell in the family (right). The bar plots below show the percent total and recurrent PB families with a given isotype. **e**, PBs were isolated from D1 and D2 7 d after influenza vaccination in 2010–11 and plated on mesenchymal stromal cells. The culture supernatants were tested for the presence of IgG antibodies to influenza vaccine and to recall antigens to which the individual was not recently exposed. TT, tetanus toxoid; MV, measles virus, VZV, varicella zoster virus; TG, *Toxoplasma gondii*. **f**, The same analysis was performed on PBs isolated in the steady state. **g**, The specificity of the antibodies produced by single PBs of D1 was confirmed through the isolation of monoclonal antibodies against TT or MV.

**Extended Data Fig. 8:**
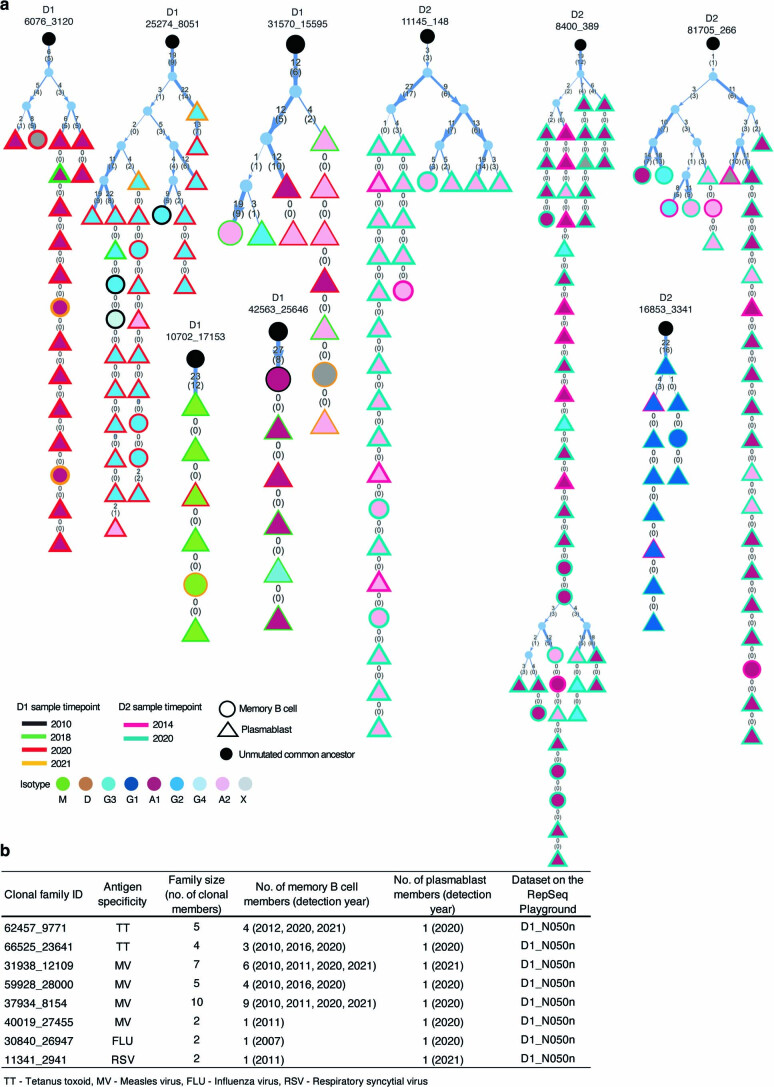
Recurrent clonotypes in circulating plasmablasts. **a**, Genealogical trees show representative clonal families in D1 and D2 containing recurrent plasmablasts (triangles) detected at the indicated time points. **b**, 10X experiments identify in D1 single plasmablasts that belong to antigen-specific families previously identifies through the isolation of monoclonal antibodies. Shown is the clonal family identity (ID), the antigenic specificity, the family size and the time at which memory B cells and plasmablasts were detected.

To search for recurrent clonotypes, we used the 10X platform to sequence four samples of plasmablasts from D1 and four from D2 obtained at different time points. In both donors several clonal families were detected at two or more time points and some even at all time points analyzed (Fig. 5a,b and examples in Extended Data Fig. 8a). A few recurrent families were overrepresented at a given time point but found at low frequencies also at distant time points (Fig. 5c,d). In each donor, the isotype composition of recurrent families was comparable to the isotype composition of total plasmablast families (Fig. 5c,d) and comprised mainly IgA and IgM, followed by IgG1 and IgG2. These findings reveal a sustained production of clonal plasmablasts that may reflect the continuous stimulation of memory B cells by environmental or persisting antigens.

To investigate whether plasmablast production from memory B cells may proceed long-term even in the absence of recent antigenic stimulation, we interrogated the 10X data from D1 and identified several plasmablasts in the 2020–2021 samples that matched the sequences of monoclonal antibodies specific for tetanus toxoid, measles virus, influenza virus or respiratory syncytial virus that were previously isolated from immortalized memory B cells from the same donor (Extended Data Fig. 8b). Notably, D1 was infected by measles virus as a child, was boosted with tetanus toxoid vaccine in 1999 and 2015 and received the last influenza vaccination in 2017. Thus, these findings suggest that plasmablasts specific for recall antigens are continually produced at low-rate months and years after antigenic stimulation.

In another experimental setting, we cultured single plasmablasts on mesenchymal stromal cells and analyzed the secreted antibodies^16^. Following a booster influenza vaccination in 2011, a large fraction of total plasmablasts (20% and 43% in D1 and D2) secreted IgG antibodies to the vaccine as expected (Fig. 5e). Of note, however, rare plasmablasts secreting IgG antibodies that did not bind to the vaccine but bound to unrelated recall antigens (tetanus toxoid, measles virus, varicella zoster virus and *Toxoplasma gondii*) were also detected in both donors at very low frequencies **(**Fig. 5e). Plasmablasts specific for recall antigens were also detected in both donors in the steady state, a finding that was corroborated by the isolation from positive cultures of monoclonal antibodies specific for measles virus and tetanus toxoid (Fig. 5f,g).

Collectively, the identification of recurrent plasmablasts of diverse isotypes and specificities including IgG1 antibodies to recall antigens suggest that a large fraction and possibly most memory B cell families continuously produce plasmablasts in response to persisting antigens or polyclonal stimuli.

## Discussion

This study provides a high-level kinetic assessment of the entire human memory B cell and circulating plasmablast repertoires and reveals three aspects: (1) the large expansion and multi-isotype differentiation of IgM and IgA clonal families; (2) the overall stability of memory B cell and circulating plasmablast families; and (3) the sustained and recurrent production of plasmablasts derived from memory B cell families.

IgM memory B cells have been known for a long time to represent a large and distinct fraction of the human memory pool^17–20^. Our analysis demonstrates that IgM comprises very large and stable clonal families that reach up to 0.3% of total memory B cells and show a frequent intra-clonal switch to IgA and IgG2. The relatively small size of IgG1 B cell families specific for recall antigens suggest that the large IgM IgA and IgG2 clonal families may recognize a different class of antigens such as microbial pathogens or commensals that are persistent or widely accessible^21^.

Several studies showed convergent V gene usage in response to vaccines and infections^7,22–26^ and two recent reports using ultradeep sequencing of VH genes from healthy donors showed that 0.2–1.5% of sequences are shared among any two individuals^3,4^. Despite the lower depth and more stringent criteria based on VH/VL identity, our analysis comes to a similar estimate of 0.2–0.4% shared clonotypes between the two donors analyzed. These findings suggest that such convergent clonotypes may be selected by common environmental antigens, possibly with unique structural motifs^27^. The production of recombinant antibodies using the VH/VL sequences available in the datasets and RepSeq Playground will be instrumental to determine the specificity of the antibodies produced by convergent or expanded families.

Although mutations and isotype switching are both mediated by activation-induced cytidine deaminase enzyme, the two mechanisms are differentially regulated in time and space^28,29^. The genealogic analysis of large families comprising both memory B cells and plasmablasts shows multiple switching events occurring at different branches in already mutated cells as well as in cells with identical mutations. This finding indicates that switching involves both proximal and distal isotypes classes^30^ and that the option to switch remains open during or following somatic mutations.

Circulating plasmablasts are thought to reflect ongoing immune responses^31–33^, a notion that is consistent with our finding of a substantial homogeneity of the plasmablasts analyzed and with the broad distribution of clonal families within multiple UMAP clusters. Thus, it was surprising to find that at any time point a substantial fraction of plasmablasts derive from long-term memory B cells identified several years before and that certain B cell families produce plasmablasts at multiple and even at all time points analyzed. While it is possible that these recurrent plasmablasts may be produced in response to continuous stimulation by environmental, commensal or persisting antigens, it is unlikely that this mechanism may account for the sustained production of IgG antibodies to measles virus or tetanus toxoid that represent recall antigens to which the donor was exposed several years before the analysis. It is therefore tempting to speculate that sustained production of plasmablasts could be also maintained by antigen-independent mechanisms acting on memory B cells that are prone to differentiate in response to polyclonal stimuli^9,31^.

While it is well established that plasma cells can become long lived in bone marrow niches^15^, it has also been shown that bone marrow cells are heterogeneous^34,35^ and that vaccine-induced plasma cells can transiently populate the bone marrow but decrease to pre-boost levels after 1 year^36^. We suggest that recurrent plasmablast production may represents a homeostatic mechanism that counteracts the attrition of bone marrow plasma cells.

## Methods

### Donors and sample collection

Peripheral blood samples were collected from two healthy male donors, D1 (69 years old) and D2 (50 years old) at the time of latest sampling. Both D1 and D2 were vaccinated with the seasonal vaccine in year 2010–11 and 2009–10, respectively. Peripheral blood mononuclear cells (PBMCs) from the year 2010 (D1) and 2014 (D1) were isolated by standard density-gradient centrifugation and cryopreserved until the day of use. Blood samples collected in 2020 from both D1 and D2 were processed immediately to isolate memory B cells and plasmablasts by flow cytometry followed by single-cell immune profiling using the 10X system. The donors provided written informed consent for using these blood samples, following approval by the Cantonal Ethical Committee of Canton Ticino, Switzerland.

### Isolation of memory B cells and plasmablasts

The CD19^+^ cell fraction was enriched from PBMCs by positive selection with CD19 magnetic microbeads (Miltenyi Biotech) and subsequently stained on ice for 20 min with the following fluorochrome-labeled mouse monoclonal antibodies: CD3-APC/Cy7 (dilution 1:40, clone HIT3a, catalog no. 300317, BioLegend), CD27-Bv650 (dilution 1:50, clone O323, catalog no. 302827, BioLegend), CD19-PE-Cy7 (dilution 1:50, clone SJ25C1, catalog no. 341113, BD Biosciences), HLA-DR-BD Horizon V500 (dilution 1:100, clone G46-6, catalog no. 561224, BD Biosciences) and CD38-PE (dilution 1:100, clone T16, catalog no. IM1832U, Beckman Coulter). Cells were sorted to over 98% purity on a FACSAria III (BD) using the following gating strategy: circulating memory B cells were sorted as CD3^–^CD19^+^CD27^+^CD38^−/+^ cells, whereas circulating plasmablasts were sorted as CD3^–^CD19^+^CD27^hi^CD38^hi^ cells. FACS-sorted cells were collected in 6 μl FCS in Eppendorf tubes that were pre-coated overnight with 2% BSA.

### Single-cell RNA-seq library preparation and sequencing

The 5ʹ single-cell RNA-seq libraries were generated using Chromium Next GEM Single Cell V(D)J Reagent kit v.1, 1.1 or v.2 (10X Genomics) according to the manufacturer’s protocol. Paired heavy and light chain BCR libraries were prepared from the sorted B cell populations. Additional gene expression (messenger RNA) libraries were constructed from freshly isolated plasmablasts from D1. Briefly, up to 20,000 memory B cells and 5,000–10,000 plasmablasts (depending on their recovery from total B cell pool from given donor or time point) per well of 10X chip were loaded in the 10X Genomics Chromium Controller to generate single-cell gel beads in emulsion. After reverse transcription, gel beads in emulsion were disrupted. Barcoded complementary DNA was isolated and used for the preparation of BCR and gene expression libraries. All the steps were followed as per the manufacturer’s instructions in user guide recommended for 10X Genomics kit v.1, 1.1 or 2. The purified libraries from each time point were pooled separately and sequenced on the NextSeq550 (Illumina) as per the instructions provided in 10X Genomics user guide for the read length and depth. The D1 sample from February 2021 included memory B cells and plasmablasts sequenced on NovaSeq (Illumina) to obtain paired VDJ and gene expression information. Based on unique molecular identifier (UMI) (VDJ transcript) count and gene expression information, data for plasmablasts were extracted and used in the study.

### Bioinformatic analyses

We used CellRanger (v.5.0.0) pipeline for raw sequencing processing, including 5′ gene expression analysis and V(D)J analysis of memory B cells and plasmablasts. Using CellRanger, outputs following downstream analyses were performed.

#### Computational analyses of V(D)J sequences

Raw output files were demultiplexed and processed using CellRanger v.5.0.0 software (10X Genomics). For each donor, a donor-specific VJ genes database was generated using IgDiscover^10^, whereas to identify the convergent clonotypes, publicly available reference sequences from the IMGT/V-QUEST reference directory at https://www.imgt.org/ were used to jointly analyze the antibody sequences from both donors. Next, data were processed and analyzed using the Immcantation Framework (http://immcantation.org) with Change-O v.1.0.2 (ref. ^11^). For each 10X dataset, the filtered_contig.fasta file was annotated using IgBlast v.1.16 (ref. ^37^) with the related donor-specific VJ genes database. To generate adaptive immune receptor repertoire (AIRR) rearrangement data, the filtered_contig_annotations.csv file was used and only productive sequences were kept. The heavy and light chain sequences were separated in two files. The threshold for trimming the hierarchical clustering of B cell clones was determined by the SHazaM module for determining distance to nearest neighbor^11^. With the Change-O DefineClones function, clones were assigned based on IGHV genes, IGHJ gene and junction distance calculated by SHazaM (distance 0.15). The generated clone-pass file was verified and corrected using the Change-O light_cluster function, based on the analysis of the light chain partners associated with the heavy chain clone. Independent clone-pass files were generated for each 10X run. For downstream analysis, all clone-pass files from D1 and D2 donors and the D1 antigen-specific antibodies file (also processed by Change-O) were combined and re-clustered all together. Germlines were reconstructed using the Change-O CreateGermlines function. To obtain the final AIRR format file containing paired information on the same row, we used a Java script to process and filter the sequences. Only the heavy chains paired with one κ and/or one λ were filtered in for downstream analysis. Finally, matrices with percentage of identity between each sequence within each clone (heavy and light separated), were generated for the RepSeq Playground. Dnaml from Phylip package (v.3.69) or IgPhyml (optional) was used to produce the genealogical trees of each clone^14,38^. The Morisita–Horn similarity index between the different samples was calculated using the morisita.horn function from the fossil R package^39–41^. Honeycomb plots were created using the enclone tool (v.0.5) available from 10X Genomics. The class-switching propensity matrices were calculated by counting the number of clonal families containing each possible pair of immunoglobulin classes (upstream and downstream isotypes) and the families having cells of unique isotype only (diagonal).

#### Single-cell RNA-seq data quality control, processing, annotation and differential gene expression analysis

For single plasmablast transcriptome analysis, we mainly used the CellRanger pipeline^42^ and Seurat package^43^ for quantification, quality control, data normalization, dimensionality reduction, clustering, differential expression analysis and data visualization. We used the CellRanger pipeline to generate gene expression count matrices from the raw data. For each sample, a gene-by-cell counts matrix was used to create a Seurat object using Seurat^43,44^. We filtered cell barcodes with <500 and >2,500 UMIs as well as >5% mitochondrial contents. Each individual sample was then normalized by a factor of 10,000 and log transformed (NormalizeData). The top 2,000 most variable genes were then identified using the FindVariableFeatures method. The gene expression matrix obtained by applying the filtering steps above was then used to perform principal-component analysis (RunPCA), preliminary clustering analysis, including nearest neighbor graph (FindNeighbors) and unbiased clustering (FindClusters) and cell type annotation. UMAP was then used to visualize the expression data. We identified gene expression markers for each cluster using FindAllMarkers from Seurat with default settings, including Wilcoxon test and Bonferroni *P* value correction^43,44^. Differential gene expression between specified clusters (or subclusters) was performed using FindMarkers (Wilcoxon rank-sum test) with Benjamini–Hochberg false discovery rate (FDR) correction and average log fold change (FC). Genes were considered (significantly) differentially expressed if FDR < 0.05 and logFC > 0.2 in a given group. Cells with undetectable or very low expression of the specific marker genes such as *XBP1*, *PRDM1* and *TNFRSF17* were removed from the downstream analyses. All computational analyses were performed in R (v.3.6.3)

### RepSeq Playground: interactive BCR platform to interrogate B cell repertoires

RepSeq Playground (v.1.0) is a user-friendly and interactive web-based platform for the analysis and visualization of BCR sequencing data. It enables immunologists (users) to easily visualize and explore BCR data to better understand the underlying biological mechanisms in a short period of time. The platform can handle combined data from multiple sources and experiments, including screened antigen-specific antibody sequences and paired or unpaired heavy and light chain sequences obtained from high-throughput bulk or single-cell sequencing experiments. The functionalities of the platform range from simple descriptive statistical charts to more advanced analyses of clonal family expansion. For descriptive visualizations, it offers statistical comparisons between donors or different experimental datasets such as the total number (or percentage) of sequences, CDR3 length distribution, isotype distribution or gene usage. To better understand clonal expansion, it is possible to interactively visualize each clonal family with a graph structure on the platform, with each node representing a sequence and an edge corresponding to a homology similarity between a pair of sequences. When the end user hovers the mouse over nodes and edges, the platform provides detailed information on each sequence and homology similarity. RepSeq Playground also generates phylogenetic tree analysis for four different methods. In addition, it provides multiple sequence alignment for each clonal family and further annotations based on various input factors such as isotype, gene usage, antigen specificity and collection date. RepSeq Playground is accessible at https://repseq.bigomics.ch to interactively visualize and analyze individual clonal families from D1 and D2.

### Monoclonal antibody isolation from memory B cells

Memory B cells were isolated from cryopreserved or fresh PBMCs using FITC-labeled anti-CD22 monoclonal antibody (BD Biosciences) followed by anti FITC-beads (Miltenyi) and cell sorting on a FACSAria (BD Biosciences). Cells were immortalized with Epstein–Barr virus (EBV) as described previously^45^. After 2 weeks, culture supernatants were screened for the presence of monoclonal antibodies specific to recall antigen (measles virus, tetanus toxoid, influenza virus and respiratory syncytial virus) and positive EBV B cell cultures were expanded in complete RPMI medium. VH and VL sequences were obtained from positive B cell cultures by PCR with reverse transcription (RT–PCR) and antibodies were produced by transfection of HEK293T cells to formally prove their specificity as described^26^.

### Analysis of antibodies produced by plasmablasts against recall antigens

Recently generated circulating plasmablasts were isolated form healthy donors in the steady state or 7 d after intramuscular vaccination with seasonal influenza vaccine. Briefly, plasmablasts were isolated from PBMCs using CD138-PE and anti-PE magnetic beads, followed by staining with HLA-DR-APC and L-selectin-PC5 and cell sorting on a FACSAria (BD). CD138^+^HLA-DR^+^L-selectin^+^ circulating plasmablasts were seeded on a monolayer of mesenchymal stromal cells at 0.5 cells per well in 384 well plates in RPMI 1640 medium supplemented with 10% FCS (Hyclone) and 10 ng ml^−1^ human r-interleukin-6 (R&D)^16^. After 3 d, culture supernatants were collected using an automated liquid handling equipment (PerkinElmer) and tested in micro-ELISA (5 µl per well) for the presence of IgG antibodies to the specific antigens. From positive cultures, the VH and VL sequences were retrieved by nested RT–PCR and cloned into human IgG1 and IgL expression vectors (kindly provided by M. Nussenzweig, Rockefeller University) as described^46^. Monoclonal antibodies were produced by transient transfection of 293 Freestyle cells (Invitrogen) with polyethyleneimine. Supernatants from transfected cells were collected after 7 d of culture.

### ELISA

To screen plasma cell supernatants, high protein binding 384 well plates (PerkinElmer) were coated with 1 µg ml^−1^ of tetanus toxoid, measle virus, varicella zoster virus, *Toxoplasma* *gondii* antigens or 1:100 influenza vaccine preparation in PBS. After blocking with PBS 1% BSA, culture supernatants were added for 1 h at room temperature. Plates were then washed and alkaline phosphatase-conjugated F(abʹ)_2_ goat anti-human IgG (Southern Biotech) was added for further 45 min at room temperature. Antibody binding was revealed, after washing plates, by adding substrate (p-NPP, Sigma). Plates were read at 405 nm.

### Statistical analysis

Flow cytometric data were acquired using BD FACSDiva (v.9.0) software. Flow cytometric data were analyzed using FlowJo (v.10.7.1) and CellRanger 5.0 pipeline for the preprocessing of raw V(D)J and gene expression data. Single-cell RNA-seq data were analyzed using R (v.3.6.3) and Seurat (v.4). R (v.3.6.3) and GraphPad (v.9.3.1, Prism software) were used to perform graphing and statistical analyses. No statistical methods were used to predetermine sample size. The experiments were not randomized and investigators were not blinded to allocation during experiments and outcome assessment.

### Reporting summary

Further information on research design is available in the Nature Research Reporting Summary linked to this article.

## Online content

Any methods, additional references, Nature Research reporting summaries, source data, extended data, supplementary information, acknowledgements, peer review information; details of author contributions and competing interests; and statements of data and code availability are available at 10.1038/s41590-022-01230-1.

## Supplementary information

Reporting Summary

## Data Availability

All single B cell 10X VDJ generated in this study are available under ArrayExpress accession E-MTAB-11174 and E-MTAB-11697. Single-cell 5ʹ gene expression data of plasmablasts are available at the NCBI Gene Expression Omnibus database: GSE188681. Antigen-specific single-cell HC and LC sequences are available from GenBank under accession numbers OL450601–OL451038. The authors declare that all data supporting the findings of this study are available within the article and its supplementary files can be obtained from the corresponding authors upon reasonable request.

## Code availability

The code for preprocessing BCR sequences is available at https://github.com/MathildeFogPerez/manuscript-rep-phad.

RepSeq Playground (v.1.0) is accessible at https://repseq.bigomics.ch.
