# Supplementary material for: Clonal structure, stability and dynamics of human memory B cells and circulating plasmablasts
Source: Nat Immunol. 2022 Jun 27;23(7):1076–85. doi: 10.1038/s41590-022-01230-1 (PMC9276532; doi:10.1038/s41590-022-01230-1)
Supplement: Supplementary file 1 — Reporting Summary [file 41590_2022_1230_MOESM1_ESM.pdf]

Corresponding author(s): Prof. Antonio Lanzavecchia, Dr. Ganesh E. Phad

Last updated by author(s): Apr 25, 2022

Reporting Summary

Nature Portfolio wishes to improve the reproducibility of the work that we publish. This form provides structure for consistency and transparency in reporting. For further information on Nature Portfolio policies, see our Editorial Policies and the Editorial Policy Checklist.

Statistics

For all statistical analyses, confirm that the following items are present in the figure legend, table legend, main text, or Methods section.

n/a	Confirmed	
☐	☒	The exact sample size (*n*) for each experimental group/condition, given as a discrete number and unit of measurement
☐	☒	A statement on whether measurements were taken from distinct samples or whether the same sample was measured repeatedly
☐	☒	The statistical test(s) used AND whether they are one- or two-sided<br>*Only common tests should be described solely by name; describe more complex techniques in the Methods section.*
☒	☐	A description of all covariates tested
☒	☐	A description of any assumptions or corrections, such as tests of normality and adjustment for multiple comparisons
☐	☒	A full description of the statistical parameters including central tendency (e.g. means) or other basic estimates (e.g. regression coefficient) AND variation (e.g. standard deviation) or associated estimates of uncertainty (e.g. confidence intervals)
☐	☒	For null hypothesis testing, the test statistic (e.g. *F*, *t*, *r*) with confidence intervals, effect sizes, degrees of freedom and *P* value noted<br>*Give P values as exact values whenever suitable.*
☒	☐	For Bayesian analysis, information on the choice of priors and Markov chain Monte Carlo settings
☒	☐	For hierarchical and complex designs, identification of the appropriate level for tests and full reporting of outcomes
☒	☐	Estimates of effect sizes (e.g. Cohen's *d*, Pearson's *r*), indicating how they were calculated

Our web collection on statistics for biologists contains articles on many of the points above.

Software and code

Policy information about availability of computer code

Data collection

FACS Diva v9.0
NextSeq550 (Illumina)
NovaSeq (Illumina)

Data analysis

BD FACSDiva (v9.0) to acquire flow cytometric data.
Flowjo (v10.7.1) to analyze flow cytometric data.
Cellranger 5.0 pipeline for the preprocessing of raw V(D)J and the gene expression data.
IgDiscover (v0.12.3) (igdiscover.se) to find individual-specific antibody-encoding germline genes.
IgBlast (version 1.16) used for the gene assignment to the sequences
Change-O (v1.0.2) (http://immcantation.org) for clonal grouping
Phylip package (v3.69) was used to produce the genealogical trees of each clonal family.
Code for pre-processing BCR sequences is available at https://github.com/MathildeFogPerez/manuscript-rep-phad
Seurat (v4) for the gene expression analysis of plasma cells.
enclone (v0.5) to create honeycomb plots.
RepSeq Playground (v1.0) (https://repseq.bigomics.ch) to interactively visualize and analyze individual clonal families.
R (v3.6.3) used for the data processing, graphing and statistical analysis.
GraphPad Prism 9 was used to perform graphing and statistical analyses.

For manuscripts utilizing custom algorithms or software that are central to the research but not yet described in published literature, software must be made available to editors and reviewers. We strongly encourage code deposition in a community repository (e.g. GitHub). See the Nature Portfolio guidelines for submitting code & software for further information.

Data

Policy information about availability of data

All manuscripts must include a data availability statement. This statement should provide the following information, where applicable:

- Accession codes, unique identifiers, or web links for publicly available datasets
- A description of any restrictions on data availability
- For clinical datasets or third party data, please ensure that the statement adheres to our policy

FACS gating strategies are indicated in the methods section. All single B cell 10X VDJ generated in this study are available under ArrayExpress accession E-MTAB-11174 and E-MTAB-11697. Single cell 5' gene expression data of plasma cells are available at the NCBI Gene Expression Omnibus database: GSE188681. Antigen-specific single-cell HC and LC sequences are available from GenBank under accession numbers OL450601 - OL451038. The authors declare that all data supporting the findings of this study are available within the article and its supplemental files or can be obtained from the corresponding authors upon reasonable request.

Field-specific reporting

Please select the one below that is the best fit for your research. If you are not sure, read the appropriate sections before making your selection.

☒ Life sciences ☐ Behavioural & social sciences ☐ Ecological, evolutionary & environmental sciences

For a reference copy of the document with all sections, see nature.com/documents/nr-reporting-summary-flat.pdf

Life sciences study design

All studies must disclose on these points even when the disclosure is negative.

Sample size	No statistical methods were used to predetermine sample size. Multiple samples from two healthy adult male donors with an interval of 6-10 years were collected and analyzed as described in the text. The sample type and collection date information is provided in the Extended Data Fig. 1.
Data exclusions	Cells with undetectable or very low expression of the specific marker genes such as XBP1, PRDM1, and TNFRSF17 from plasma cell population were removed from the downstream analyses. No other data were excluded.
Replication	This study includes the analyses of longitudinal samples collected several years apart from two healthy donors. The samples collected at different time points are unique to the donor; therefore, replicating experiments was not possible. The samples from each time point and donor were processed and profiled on separate days to avoid cross-contamination. The overall findings are comparable between both donors.
Randomization	N/A. Healthy donors included in the study were not divided into experimental groups.
Blinding	N/A. Blinding was not relevant to this study, as there was no group allocation for the donors.

Reporting for specific materials, systems and methods

We require information from authors about some types of materials, experimental systems and methods used in many studies. Here, indicate whether each material, system or method listed is relevant to your study. If you are not sure if a list item applies to your research, read the appropriate section before selecting a response.

Materials & experimental systems

n/a	Involved in the study	
☐	☒	Antibodies
☐	☒	Eukaryotic cell lines
☒	☐	Palaeontology and archaeology
☒	☐	Animals and other organisms
☐	☒	Human research participants
☒	☐	Clinical data
☒	☐	Dual use research of concern

Methods

n/a	Involved in the study	
☒	☐	ChIP-seq
☐	☒	Flow cytometry
☒	☐	MRI-based neuroimaging

Antibodies

Antibodies used	CD3-APC/Cy7 (dilution 1:40, clone HIT3a, catalog no. 300317), CD27-Bv650 (dilution 1:50, clone O323, catalog no. 302827) from BioLegend, CD19-PE-Cy7 (dilution 1:50, clone SJ25C1, catalog no. 341113) , HLA-DR-BD Horizon V500 (dilution 1:100, clone G46-6, catalog no. 561224) from BD Biosciences, CD38-PE (dilution 1:100, clone T16, catalog no. IM1832U) from Beckman Coulter.

Validation	Reactivity of above mentioned antibodies is based on the information on manufacturer's homepages

Eukaryotic cell lines

Policy information about cell lines

Cell line source(s)	293FT (Thermo Fisher, R70007); EXPI293 (Thermo Fisher, A14527)
Authentication	These cell lines were obtained from vendors that sell authenticated cell lines, they grew, performed and showed morphology as expected. No additional specific authentication was performed.
Mycoplasma contamination	Cell lines are routinely tested and tested negative for mycoplasma.
Commonly misidentified lines (See ICLAC register)	No commonly misidentified cell lines were used in this study.

Human research participants

Policy information about studies involving human research participants

Population characteristics	Two healthy adult male donors (69 yrs and 50 yrs old) sampled longitudinally. The information of sample type and collection date is provided in Extended Data Fig. 1.
Recruitment	Healthy donors were included in the study and sampled longitudinally with an interval of 10 years (in the year 2010 and 2020) and 6 years (in the year 2014 and 2020), respectively. During the samplings, both donors had no history of recent vaccination or infection.
Ethics oversight	The study protocols were approved by Cantonal Ethics Committee of Ticino, Switzerland (CE-TI-3428, 2018-02166; CE-TI-3687, 2020-01572)

Note that full information on the approval of the study protocol must also be provided in the manuscript.

Flow Cytometry

Plots

Confirm that:

- [x] The axis labels state the marker and fluorochrome used (e.g. CD4-FITC).
- [x] The axis scales are clearly visible. Include numbers along axes only for bottom left plot of group (a 'group' is an analysis of identical markers).
- [x] All plots are contour plots with outliers or pseudocolor plots.
- [ ] A numerical value for number of cells or percentage (with statistics) is provided.

Methodology

Sample preparation	No tissue processing was performed, thus cells were stained in suspension, as written in protocol.
Instrument	BD Aria
Software	BD FlowJo (v10.7.1), BD FACSDiva (v9.0)
Cell population abundance	Samples were sorted based on 4-way purity setting and no post-sort validation was performed.
Gating strategy	Gating on live cells was performed using FSC and SSC, followed by single cell gating.

- [x] Tick this box to confirm that a figure exemplifying the gating strategy is provided in the Supplementary Information.
